# Cross-Sectional Associations between Body Mass Index and Hyperlipidemia among Adults in Northeastern China

**DOI:** 10.3390/ijerph13050516

**Published:** 2016-05-20

**Authors:** Wenwang Rao, Yingying Su, Guang Yang, Yue Ma, Rui Liu, Shangchao Zhang, Shibin Wang, Yingli Fu, Changgui Kou, Yaqin Yu, Qiong Yu

**Affiliations:** 1 Department of Epidemiology and Biostatistics, School of Public Health, Jilin University, Changchun 130021, China; raoww14@mails.jlu.edu.cn (W.R.); suyy14@mails.jlu.edu.cn (Y.S.); 5143086676@163.com (G.Y.); mayue205@163.com (Y.M.); ruiliu15@mails.jlu.edu.cn (R.L.); zhangsc15@mails.jlu.edu.cn (S.Z.); spiriorwang@126.com (S.W.); fuyingli318@126.com(Y.F.); koucg@jlu.edu.cn(C.K.); yuyaqin5540@163.com (Y.Y.); 2 Unit of Psychiatry, Faculty of Health Sciences, University of Macau, Macao SAR 999078, China

**Keywords:** hyperlipidemia, BMI, relationship

## Abstract

*Background*: There is evidence that body mass index (BMI) is closely related to hyperlipidemia. This study aimed to estimate the cross-sectional relationship between Body Mass Index (BMI) and hyperlipidemia. *Methods*: We recruited 21,435 subjects (aged 18–79 years and residing in Jilin province, China) using the multistage stratified cluster random sampling method. Subjects were interviewed with a standardized questionnaire and physically examined. We analyzed the cross-sectional relationship between BMI and hyperlipidemia. *Results*: The prevalence of hyperlipidemia was 51.09% (52.04% in male and 50.21% in female). The prevalence of overweight and obesity was 31.89% and 6.23%, respectively. Our study showed that underweight (OR = 0.499, 95% CI: 0.426–0.585), overweight (OR = 2.587, 95% CI: 2.428–2.756), and obesity (OR = 3.614, 95% CI: 3.183–4.104) were significantly associated with hyperlipidemia (*p* < 0.001) in the age- and sex-adjusted logistic regression. After further adjusting for age, gender, region, district, ethnicity, education, marital status, main occupation, monthly family income per capita, smoking, drinking, exercise, central obesity, waist and hip, underweight (OR = 0.729, 95% CI: 0.616–0.864), overweight (OR = 1.651, 95% CI: 1.520–1.793), and obesity (OR = 1.714, 95% CI: 1.457–2.017) were independently associated with hyperlipidemia (*p* < 0.001). The restricted cubic spline model illustrated a nonlinear dose-response relationship between levels of BMI and the prevalence of hyperlipidemia (*P*_nonlinearity_ < 0.001). *Conclusion*: Our study demonstrated that the continuous variance of BMI was significantly associated with the prevalence of hyperlipidemia.

## 1. Introduction

Hyperlipidemia, a major systemic disorder, is an important modifiable risk factor for coronary heart disease and extra-coronary atherosclerosis [1,2] and associated with a higher risk of cardiovascular disease (CVD), which is a leading contributor to mortality worldwide, particularly in China [3]. Estimates from the World Health Organization (WHO) suggested that CVDs contributed to approximately 17.5 million individuals deaths in 2012 [4].

Moreover, hyperlipidemia is commonly associated with obesity which is known as a risk factor for CVD [5,6]. Converging lines of evidence suggests that hyperlipidemia is associated with a high BMI [7,8]. Around 3.4 million adults deaths, 3.9% of years of life lost and 3.8% of disability-adjusted life-years (DALYs) each year worldwide, were attributed to overweight or obese [9]. BMI is currently the most widely used anthropometric measurement to predict health risk related to weight status, and a large number of studies have reported a significant relationship between BMI and hyperlipidemia [10]. However, previous studies converted BMI into categorical variables when performing the multivariate analysis.

In the present study, we examined the nonlinear dose-response relationship between the continuous variance of BMI and the prevalence of hyperlipidemia. As an intuitive method for presenting data, the restricted cubic spline has been widely used in the field of meta-analysis and other researches [11,12,13].

## 2. Experimental Section

### 2.1. Study Population

Our study was conducted in the framework of the Project on Present Situation and Change Forecast of Disease Spectrum in Jilin Province, China. It was supported by the Bureau of Public Health of Jilin Province, China. A total of 21,435 participants (community-dwelling residents aged 18 to 79 years) were enrolled from June 2012 to August 2012 and completed the survey. Subjects with incomplete blood lipid information were excluded. Our study adopted a multistage stratified cluster random sampling method, with the sample selected from all nine administrative regions in Jilin Province. The details of the sampling process were published elsewhere [14].

We stuck to the bioethics principles of the Declaration of Helsinki, and our study was authorized by the Ethics Committee of Jilin University School of Public Health (Reference Number: 2012-R-011) and the Bureau of Public Health of Jilin Province(Reference Number: 2012-10). All participants voluntarily joined this study with informed consents.

### 2.2. Data Collection and Measurements

Our study adopted a structured pre-coded personal health survey questionnaire compiled by the Bureau of Public Health of Jilin Province united with the School of Public Health of Jilin University and the Jilin Disease Prevention and Control Information Platform. Participants were interviewed in a face-to-face manner by specially trained researchers. The collected information included demographic characteristics (region, age, gender, level of education and main occupation), lifestyles (smoking, drinking and exercise), history of hyperlipidemia in the past one year, and current treatment of hyperlipidemia.

In the light of the standard protocols and techniques, the participants went through anthropometric examinations including height, weight, waist, and hip circumference measurements by a trained certified research practitioner. Participants with light-colored clothing were measured for their weight as well as waist and hip circumference early in the morning after the food-intake. They were measured for heights after taking shoes off. Each physical measurement was completed by two research assistants.

Fasting venous blood samples of each participant were extracted by venipuncture for measuring the levels of blood lipids, including total cholesterol (TC), high-density lipoprotein cholesterol (HDL-C), low-density lipoprotein cholesterol (LDL-C), and triglycerides (TG). Blood lipids were measured using the MODULE P800 automated biochemistry analyzer (ROCHE Diagnostics Ltd., Indianapoils, IN, USA) in a core laboratory with a standard protocol.

### 2.3. Definitions

In terms of the Chinese guidelines on the prevention and treatment of dyslipidemia in adults (2007) [15], hyperlipidemia was defined as total cholesterol (TC ≥ 5.18 mmol/L), and/or low-density lipoprotein cholesterol (LDL-C ≥ 3.37 mmol/L), and/or high-density lipoprotein cholesterol (HDL-C < 1.04 mmol/L), and/or triglycerides (TG ≥ 1.70 mmol/L), and/or with history of hyperlipidemia diseases in the past one year. BMI was defined as a person’s weight in kilograms divided by the square of his/her height in meters (kg/m^2^). Our study categorized adults as: Underweight with BMI < 18.5 kg/m^2^,normal weight with 18.5 ≤ BMI < 24 kg/m^2^, overweight with 24 ≤ BMI < 30 kg/m^2^,and obese with BMI ≥ 30 kg/m^2^ [16]. Central obesity was defined as the waist circumference ≥85 cm for man or ≥80 cm for woman [17]. The smoking status was categorized into current smoker (smoking at least 100 cigarettes in their lifetime and smoking daily or during the time of the survey), former smoker (smoking at least 100 cigarettes in their lifetime but not smoking at the time of the survey),and non-smoking (never smoked or smoked less than 100 cigarettes in their lifetime) [18]. A drinker was defined as a person who consumed more than one alcoholic drink weekly on average, either spirits, beer, wine, or other forms of alcohol. Participants who exercised one or two times a week were classified as “sometimes exercise”; those who exercised more than three times a week were classified as “often exercise”; and those who do not or seldom exercise were classified as “never or rarely exercise”. Psychological distress was evaluated using the 12-item general health questionnaire (GHQ-12) which has been widely used in China [19]. All participants were sorted into two groups with a cut-point of 4 on a 0–12 point with those scoring 4 or more being deemed as being in psychological distress [20].

### 2.4. Statistical Analysis

For database management and statistical analysis, we used the Epidata software (version 3.1, Odense, Denmark) and the SPSS software (version 21.0, IBM SPSS, IBM Corp., Armonk, NY, USA). The Kolmogorov-Smirnov test was used to analyze for normality. The distributions of demographic characteristics between participants with and without hyperlipidemia were calculated by Chi-square tests for categorical variables or computed by the Wilcoxon signed-rank test for continuous variables if not following normal distributions. We used the adjusted logistic regression model to individually analyze the correlations of BMI and hyperlipidemia and the restricted cubic spline method was evaluated a potential non-linear relationship between BMI and hyperlipidemia [21,22,23]. Restricted cubic spline was implemented by the Stata software (version 12.0, Stata Press, College Station, TX, USA).Variables that were statistically significant at *p* < 0.05 level were entered into multivariate logistic regression analyses. All statistical tests were two-tailed and *p* < 0.05 was considered statistically significant.

## 3. Results

The prevalence of hyperlipidemia among residents on Jilin Province was 51.09% (10,951/21,435), with 52.04% in males and 50.21% in females. The prevalences of overweight and obesity were respectively 31.89% and 6.23%, with a slightly higher prevalence of both overweight and obesity in women than men (overweight:16.10% *vs.* 15.78%; obesity:3.28% *vs.* 2.95%).

Table 1 shows demographic characteristics of participants. Hyperlipidemia was significantly correlated with gender (*p* = 0.007), region (*p* < 0.001), district (*p* < 0.001), ethnicity (*p* = 0.047), education (*p* < 0.001), marital status (*p* < 0.001), main occupation (*p* < 0.001), monthly family income per capita (*p* < 0.001), smoking (*p* < 0.001), drinking (*p* < 0.001), exercise (*p* < 0.001), and central obesity (*p* < 0.001). However, subjects with or without hyperlipidemia did not show significant differences in family history of hyperlipidemia and GHQ-12 (*p* > 0.05). Table 2 presents that the age, BMI, and waist and hip circumferences of participants with hyperlipidemia were significant higher than those without hyperlipidemia (all *p* < 0.001).

As shown in Table 3, BMI levels were significantly associated with hyperlipidemia via unadjusted logistic regression (*p* < 0.001). In the age- and gender-adjusted logistic regression, our survey observed that there was a significant correlation between different BMI ranges and hyperlipidemia (*p* < 0.001). Underweight (OR = 0.729, 95% CI: 0.616–0.864) was associated with a lower prevalence of hyperlipidemia and overweight (OR = 1.651, 95% CI: 1.520–1.793), and obesity (OR = 1.714, 95% CI: 1.457–2.017) were independently associated with increased risks of hyperlipidemia (*p* < 0.001) after adjustment for age, gender, region, district, ethnicity, education, marital status, main occupation, monthly family income per capita, smoking, drinking, exercise, central obesity, and waist and hip circumferences. In further analyses, we found a significant trend between BMI and the presence of hyperlipidemia in the univariate model, model I (adjusted age and gender), and model II (adjusted age, gender, region, district, ethnicity, education, marital status, main occupation, monthly family income per capita, smoking, drinking, exercise, central obesity, and waist and hip circumferences) (all *p* < 0.001).

**Figure 1:**
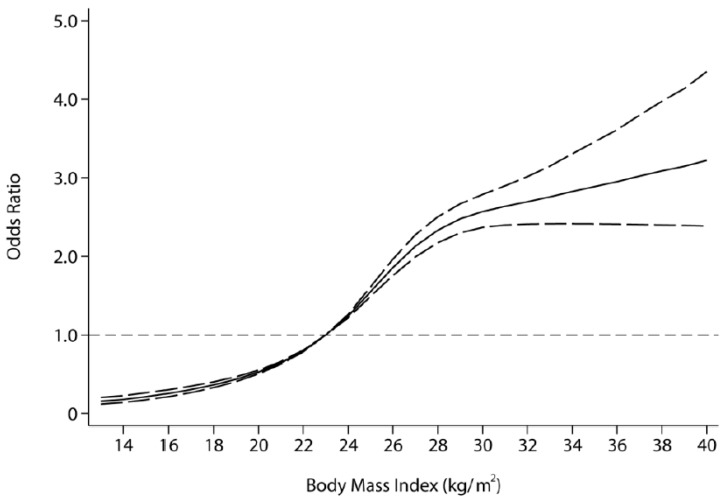
The dose-response relationship of BMI and hyperlipidemia based on the restricted cubic spline model (BMI was coded using an RCS function with four knots located at the 17.71 kg/m^2^, 22.50 kg/m^2^, 26.83 kg/m^2^ and 31.52 kg/m^2^, which respectively correspond to four sections of the median BMI. Lines with long dashes represent the pointwise 95% confidence intervals, comparing for solid lines representing the fitted nonlinear trend).

The fitted dose-response relationship is depicted in Figure 1. Overall, we found a significant nonlinear dose-response association between BMI and risk of hyperlipidemia (*p* value for nonlinearity <0.001) with a significantly increased trend of odds ratio as per 1 kg/m^2^ increase in BMI, adjusted for age, waist and hip circumferences, gender, ethnicity, education, smoking, drinking. When compared with the reference (median level of the normal BMI range, approximately BMI = 23 kg/m^2^), the ORs (95% CI) for hyperlipidemia risks were 0.36 (0.33–0.40) for BMI at 18 kg/m^2^, 1.55 (1.49–1.61) for BMI at 25 kg/m^2^, and 2.57 (2.37–2.79) for BMI at 30 kg/m^2^, indicating a significant and progressive risk of hyperlipidemia along with BMI increases.

## 4. Discussion

Our study showed that there was a significant correlation between different BMI ranges and hyperlipidemia (*p* < 0.001). Overweight (OR = 1.651, 95% CI: 1.520–1.793), and obesity (OR = 1.714, 95% CI: 1.457–2.017) were independently associated with increased risks of hyperlipidemia (*p* < 0.001) after adjustment for confounding factors. Furthermore, the dose–response analysis indicated a significant nonlinear association between BMI and the risk of hyperlipidemia, with a significantly increased trend of odds ratio as per 1 kg/m^2^ increase in BMI. The ORs (95% CI) for hyperlipidemia risks were 0.36 (0.33–0.40) for BMI at 18 kg/m^2^, 1.55 (1.49–1.61) for BMI at 25 kg/m^2^, and 2.57 (2.37–2.79) for BMI at 30 kg/m^2^, indicating a significant and progressive risk of hyperlipidemia along with BMI increases.

To our knowledge, this is the first report concerning the prevalence of hyperlipidemia in Jilin Province, northeast China. The prevalence of hyperlipidemia estimated by our study (total: 51.09%; females: 50.21%; males: 52.04%) was comparable to figures reported in published studies [24]. A previous study using data from administrative officers, who took part in annual regular physical examination from 1 September to 30 November in 1999, reported that the prevalence of hyperlipidemia in Shanghai among participants was 28.9% [25]. Considering that Shanghai is the biggest economic center of China, and one of the richest regions in China, the results of the study conducted with subjects from Shanghai may not be consistent with those from other studies. Compared to studies that examined the prevalence of hyperlipidemia among subjects from the Southwestern China, our results were similar with that reported by Deng *et al.* [26] (the prevalence of hyperlipidemia: 49.3%), but contradicted with the result of the study conducted by Yin *et al.* [27], who reported that the prevalence of hyperlipidemia was 35.91% in the Guangxi Zhuang Autonomous Region. Due to the inequality of development and large differences in lifestyles among subjects from different regions of China, the prevalence of hyperlipidemia may have contradictory results. Given that the diet of Chinese people in northeast China, with high salt and high fat content, is different from other parts of the Chinese population, our findings will be meaningful, and enable us to provide a theoretical basis when the government departments make relevant policies.

As we know, modification of excessive body weight (BMI of 27 or higher) is associated with a decreased risk of hyperlipidemia [28]. However, it is unclear whether the relationship between BMI and hyperlipidemia presented in a nonlinear trend, such as a U shaped pattern which was recognized between sleep duration and the risk of type 2 diabetes [29]. The present study demonstrated a nonlinear dose-response relationship between BMI and hyperlipidemia. The dose-response relationship has been reported in many studies [30]. For example, Friedrich *et al.* [31] reporteda U-shaped relationship between serum ferritin levels and the risk of CVD as well as IHD in women. Weiner *et al.* [32] also reported such a relationship between each of the non-traditional risk factor and outcomes using restricted cubic splines with four knots generated using S-Plus.

With the rapid economic development and urbanization process in China, the Chinese people have been experiencing tremendous nutritional transition and a big change in lifestyles [33]. Meanwhile, serum total cholesterol (TC) and low-density lipoprotein (LDL) levels of the Chinese peoplehave gradually increased in the past 10 years [34]. Therefore, our findings may have public health implications. Since obesity (BMI is 30 or higher) is an independent risk factor of hyperlipidemia, the intervention for obesity is very necessary for the Chinese people. It is well-known that healthy diets and physical activities are key to controlling the occurrence of obesity and hyperlipidemia. Therefore, we should adopt necessary measures such as replacing trans fats with unsaturated fats, implementing public awareness programs on diet and physical activity, and increasing the consumption of fruits and vegetables, to reduce trends in BMI and serum cholesterol. Furthermore, the Chinese government should pay attention to lipid-related diseases, increase investment for obesity and hyperlipidemia research, strengthen the primary health care system, inform the harm of the lipid-related disease and perform hyperlipidemia prevention and treatment [35].

Our study was subject to the following potential limitations. First, the cross-sectional nature of our study design may not allow us draw definite conclusions about a cause-and-effect relationship. However, since lifestyle behaviors and demographic characteristics for our respondents were relatively stable, our results should be considered to be reliable for policy-making. Second, due to the fact that our study did not take into account the study’s complex sampling design, our samples were not representative of adults (aged 18 to 79 years) of Jilin Province in 2012. Therefore, our results merely reflected the relationship between BMI and hyperlipidemia. Finally, our study design adapted the WHO standard of BMI classification rather than the Chinese standard, which enables us to compare with the results of other studies.

## 5. Conclusions

The results from the present study suggest that the continuous variance of BMI is significantly associated with the prevalence of hyperlipidemia. Particularly, our study indicates a dose-response nonlinear relationship between BMI and hyperlipidemia, with a significantly increased trend of odds ratio as per 1 kg/m^2^ increase in BMI. Our findings may guide the government in developing approaches to prevent hyperlipidemia and improving the healthy lifestyles of people.

## Figures and Tables

**Table 1:** Demographic characteristics of adult samples (*n* = 21,435).

Variable	*n*	Hyperlipidemia		Non-Hyperlipidemia		*χ*^2^	*p*
		*n*	%	*n*	%		
Gender						7.166	0.007
Female	11,098	5572	50.9	5526	52.7		
Male	10,337	5379	49.1	4958	47.3		
Region						18.788	<0.001
Urban	11,152	5539	50.6	5613	53.5		
Rural	10,283	5412	49.4	4871	46.5		
District						136.344	<0.001
Middle	13,322	6433	58.7	6889	65.7		
East	4104	2171	19.8	1933	18.4		
West	4009	2347	21.4	1662	15.9		
Ethnicity						3.950	0.047
Han	19,865	10,111	92.3	9754	93.0		
Other	1570	840	7.7	730	7.0		
Education						134.481	<0.001
Primary school or below	6236	3440	31.4	2796	26.7		
Junior high school	6125	3069	28.0	3056	29.1		
Senior high school	5559	2921	26.7	2638	25.2		
College and above	3515	1521	13.9	1994	19.0		
Marital status						455.081	<0.001
Married or cohabit	18,316	9613	87.8	8703	83.0		
Never married	1693	470	4.3	1223	11.7		
Divorced	388	201	1.8	187	1.8		
Widowed	1038	667	6.1	371	3.5		
Main occupation						425.563	<0.001
Unemployed	2653	1482	13.5	1171	11.2		
Mental workers	4369	1973	18.0	2396	22.9		
Manual workers	12,046	5858	53.5	6188	59.0		
Retired	2367	1638	15.0	729	7.0		
Average monthly earnings ^a^						59.992	<0.001
<500	4304	2350	21.5	1954	18.6		
500~	3959	2059	18.8	1900	18.1		
1000~	7049	3628	33.1	3421	32.6		
2000~	3983	1945	17.8	2038	19.4		
3000~	2140	969	8.8	1171	11.2		
Smoking						99.722	<0.001
Never smoked	12,992	6307	57.6	6685	63.8		
Former smoker	6723	3628	33.1	3095	29.5		
Current smoker	1720	1016	9.3	704	6.7		
Drinking						15.127	<0.001
No	14,607	7330	66.9	7277	69.4		
Yes	6828	3621	33.1	3207	30.6		
Exercise						165.482	<0.001
Often	6386	3689	33.7	2697	25.7		
Sometimes	5220	2467	22.5	2753	26.3		
Never or rarely	9829	4795	43.8	5034	48.0		
Central obesity						1730.612	<0.001
No	10,766	3978	36.3	6788	64.7		
Yes	10,669	6973	63.7	3696	35.3		
Family history ^b^						2.202	0.138
No	20,494	10,448	95.4	10,046	95.8		
Yes	941	503	4.6	438	4.2		
GHQ-12						0.586	0.444
No distress	16,356	8380	76.5	7976	76.1		
Distress	5079	2571	23.5	2508	23.9		
Diabetes							
No	19,479	9480	86.6	9999	95.4	500.924	<0.001
Yes	1956	1471	13.4	485	4.6		
Hypertension							
No	13,924	5936	54.2	7988	76.2	1137.581	<0.001
Yes	7511	5015	45.8	2496	23.8		

^a^ means monthly family income per capita; ^b^ means family history of hyperlipidemia.

**Table 2:** Distribution of age, BMI, waist and hip circumferences between participants with and without hyperlipidemia ((*n* = 21,435), M (Q_1_–Q_3_)).

Variable	Hyperlipidemia (*n* = 10,951)	Non-Hyperlipidemia (*n* = 10,484)	Z	*p*
Age (years)	51 (42–59)	43 (33–53)	−37.421	<0.001
BMI (kg/m^2^)	25.029 (22.823–27.3995)	22.977 (20.727–25.044)	−42.684	<0.001
Waist (cm)	85.300 (79.000–92.000)	79.000 (72.000–85.000)	−46.686	<0.001
Hip (cm)	96.000 (92.000–101.000)	94.000 (89.000–97.475)	−29.716	<0.001

**Table 3:** Logistic regression analyses of the influence of BMI on hyperlipidemia prevalence.

Model	BMI	B ^c^	S.E. ^d^	Wald	*p*	OR (95% CI)
Univariate	<18.5	−0.792	0.078	104.196	<0.001	0.453 (0.389–0.527)
	18.5~					1.000
	24.0~	1.008	0.031	1025.644	<0.001	2.741 (2.577–2.916)
	30.0~	1.233	0.063	379.395	<0.001	3.430 (3.030–3.883)
					*p* value for trend *p* < 0.001	
Model I ^a^	<18.5	−0.695	0.081	73.860	<0.001	0.499 (0.426–0.585)
	18.5~					1.000
	24.0~	0.951	0.032	865.523	<0.001	2.587 (2.428–2.756)
	30.0~	1.285	0.065	393.025	<0.001	3.614 (3.183–4.104)
					*p* value for trend *p* < 0.001	
Model II ^b^	<18.5	−0.316	0.086	13.337	<0.001	0.729 (0.616–0.864)
	18.5~					1.000
	24.0~	0.501	0.042	142.217	<0.001	1.651 (1.520–1.793)
	30.0~	0.539	0.083	42.235	<0.001	1.714 (1.457–2.017)
					*p* value for trend *p* < 0.001	

^a^ Adjusted age and gender; ^b^ Adjusted age, gender, region, district, ethnicity, education, marital status, main occupation, monthly family income per capita, smoking, drinking, exercise, central obesity, waist, hip; **^c^** B represents the logistics regression coefficient; **^d^** S.E. represents the standard error.
